# Centrifuge-Less Mixed Micelle-Mediated Cloud Point Extraction-Spectrophotometric Determination of Vanadium Using 4-Nitrocatechol and Cetylpyridinium Chloride

**DOI:** 10.3390/ijms26125808

**Published:** 2025-06-17

**Authors:** Andrea Gajdošová, Petya Racheva, Denitsa Kiradzhiyska, Vidka Divarova, Antoaneta Saravanska, Jana Šandrejová, Kiril Gavazov

**Affiliations:** 1 Department of Analytical Chemistry, Institute of Chemistry, Pavol Jozef Šafárik University in Košice, SK-04154 Košice, Slovakia; andrea.gajdosova@student.upjs.sk (A.G.); jana.sandrejova@upjs.sk (J.Š.); 2 Department of Chemical Sciences, Faculty of Pharmacy, Medical University of Plovdiv, 120 Buxton Bros Str., 4004 Plovdiv, Bulgaria; petya.racheva@mu-plovdiv.bg (P.R.); denitsa.kiradzhiyska@mu-plovdiv.bg (D.K.); vidka.divarova@mu-plovdiv.bg (V.D.); antoaneta.saravanska@mu-plovdiv.bg (A.S.)

**Keywords:** vanadium (V), 1,2-dihydroxy-4-nitrobenzene, centrifuge-less cloud point extraction, mixed micelle system, spectrophotometric determination

## Abstract

A novel, environmentally friendly cloud point extraction (CPE) method based on 4-nitrocatechol (H_2_L) was developed in this study to spectrophotometrically determine trace vanadium. This method utilizes a mixed micelle-mediated system comprising a cationic surfactant (cetylpyridinium chloride, CPC) and a nonionic surfactant (Triton X-114). In contrast to conventional CPE, the present approach does not employ centrifugation to separate the two phases. The distinguishing characteristic of the extracted species, (CP^+^)[V^V^OL_2_], is its ability to absorb light across the entire visible spectrum. The measurement at 670 nm, where the complex displays a local maximum, is advantageous for two primary reasons. Firstly, the blank exhibits virtually no absorption, a property that engenders stable and reproducible results. Secondly, selectivity is high because almost all other metal complexes have absorption bands at shorter wavelengths. The proposed method has the following characteristics: a linear range of 2–305 ng mL^−1^, a limit of detection of 0.6 ng mL^−1^, a molar absorptivity coefficient of 1.22 × 10^5^ M^−1^ cm^−1^, a Sandell sensitivity of 0.42 ng cm^−2^, and a blue applicability grade index (BAGI) of 67.5. Its efficacy was demonstrated in the analysis of mineral water, a spent vanadium-containing catalyst, and a dietary supplement.

## 1. Introduction

Vanadium is a dispersed trace element identified in 283 minerals [1], with an average content of 97 μg g^−1^ in the upper continental crust [2]. It is essential for various terrestrial and aquatic organisms and is considered the second most abundant transition metal in seawater [3]. Vanadium’s entry into the biosphere occurs through natural processes and anthropogenic (human-related) activities such as the combustion of fossil fuels, oil refinement, mining, ore processing, sulfuric acid production, agricultural chemicalization, and the manufacturing and utilization of various materials (steel, rubber, plastics, textiles, and paper). A notable increase in its Anthropogenic Enrichment Factor (AEF), a metric used in environmental and pollution studies, has been observed in recent years [4,5,6]. Given the toxic properties of its most common oxidation state (+V), the element is appropriately characterized as a “re-emerging environmental hazard” that we must address [6].

From another perspective, it has been demonstrated that vanadium exerts beneficial effects on human health. Vanadium-containing compounds are commercially available as dietary supplements for bodybuilders and athletes [7,8]. A substantial body of research has proven its efficacy in enhancing glucose metabolism in patients diagnosed with type 2 diabetes [9,10]. Vanadium has demonstrated anticancer properties, which may be attributable to its capacity to induce apoptosis (programmed cell death) in cancerous cells [11,12,13]. However, the balance between benefits and potential adverse effects remains to be fully elucidated. This underscores the necessity for the rigorous monitoring of patients’ conditions [7] and the development of suitable methodologies for vanadium determination in diverse samples.

A multitude of techniques have been utilized to ascertain the vanadium content. These include, but are not limited to, flame atomic absorption spectrometry [14,15,16], graphite furnace atomic absorption spectrometry [17,18,19,20,21,22,23], and inductively coupled plasma optical emission spectrometry [24]. Other techniques are inductively coupled plasma mass spectrometry [25], voltammetry [26], neutron activation analysis [27,28], and UV-vis spectrophotometry [29,30]. Spectrophotometry merits particular attention due to its operational simplicity, broad accessibility of instrumentation, cost-effectiveness, and energy efficiency. When utilized in conjunction with extraction methodologies, its performance is further enhanced, yielding improved sensitivity and selectivity across a wide range of applications.

In recent years, a significant number of chromogenic chelating reagents have been proposed for the spectrophotometric determination of vanadium following different types of extraction preconcentration. Examples include azo dyes [31,32,33,34,35,36], *N*-benzoyl-*N*-phenylhydroxylamine [37], 4-(2′,3′,4′-trihydroxyphenyl)-3-nitro-5-sulfoazobenzene [38], tannic acid [39], and thiophenols [40,41]. In the category of simple reagents with OH groups located in the ortho position, the following compounds warrant particular attention: 2,3-dihydroxynaphthalene [42], pyrogallol [43], and 4-nitrocatechol (4NC) [44,45]. Given the prominence of green technologies in contemporary analytical chemistry, cloud point extraction (CPE) is often regarded as a superior alternative to classical liquid–liquid extraction (LLE) [46,47,48,49,50]. In contrast to solid-phase extraction (SPE), which relies on costly solid sorbents or cartridges, CPE utilizes inexpensive surfactants. This technique requires only rudimentary equipment, such as a refrigerator and a hot plate, and in some variants (centrifuge-less CPE, CL-CPE), it does not even necessitate a centrifuge.

In this paper, we investigate a CL-CPE system containing 4-nitrocatechol (1,2-dihydroxy-4-nitrobenzene, 4NC) as a chelating chromophore reagent and cetylpyridinium chloride as an ion association reagent and, concomitantly, a cationic surfactant. The system also contains the nonionic surfactant Triton TX-114 (TX), which serves to enhance hydrophobic interactions. The employment of such mixed micelle (MM) systems is on the rise, as they are regarded as being particularly promising, especially when the objective is to extract charged, less hydrophobic substances [49,50].

4NC is a low-cost, widely available compound that has been identified as one of the most advantageous organic analytical reagents [51,52]. According to the CLP Regulation, the substance is classified as a non-hazardous material [53], indicating that it poses minimal risk to human health and the environment.

## 2. Results and Discussion

### 2.1. Optimization of Working Conditions

**Figure 1:**
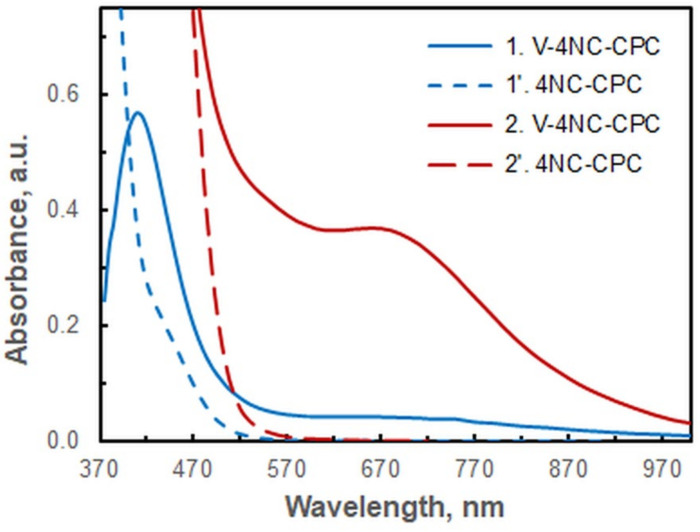
Absorption spectra of ternary complex against blank (1 and 2) and corresponding blank against water (1′ and 2′) at two different 4NC concentrations. *c*_4NC_ = 7.5 × 10^−5^ M (1 and 1′) and 7.5 × 10^−4^ M (2 and 2′); *c*_V_ = 3 × 10^−6^ M; *c*_CPC_ = 5 × 10^−4^ M; pH = 5.5; *V*_buffer_ = 2.0 mL; *w*_TX_ = 0.4%; *t*_inc_ = 70 min at 60 °C.

The spectra of the extracted species at two different 4NC concentrations (7.5 × 10^−5^ M and 7.5 × 10^−4^ M) are displayed in Figure 1. The primary absorption maximum of the vanadium complex is observed at 410 nm (Spectrum 1). Nevertheless, a wavelength of 670 nm was selected for the subsequent studies (see Spectrum 2). The utilization of this wavelength results in two notable advantages:

1. The blank sample demonstrates virtually zero absorption, a property that facilitates the experimental process (measurements are made against water) and promotes the high repeatability of the results.
2. The majority of the other 4NC complexes exhibit yellow or yellow-orange coloration (*λ* of 380–480 nm). The molar absorption coefficients of these complexes at 670 nm are negligible, a prerequisite for the development of a highly selective spectrophotometric method for determining vanadium.

**Figure 2:**
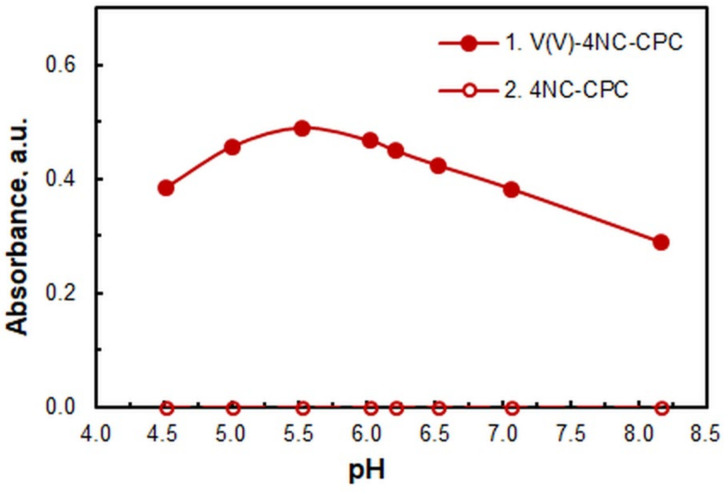
Effects of pH. *c*_V_ = 4.0 × 10^−6^ M; *c*_4NC_ = 7.5 × 10^−4^ M; *c*_CPC_ = 5 × 10^−4^ M; *w*_TX_ = 0.4%; *V*_buffer_ = 2.0 mL; *t*_inc_ = 70 min at 60 °C; λ = 670 nm.

The effects of pH on the absorbance are demonstrated in Figure 2. A series of buffer solutions, prepared from 2 M acetic acid and ammonia, was used to support the pH level. The analytical signal exhibited its maximum intensity at a pH of 5.5, a value that is regarded as optimal.

**Figure 3:**
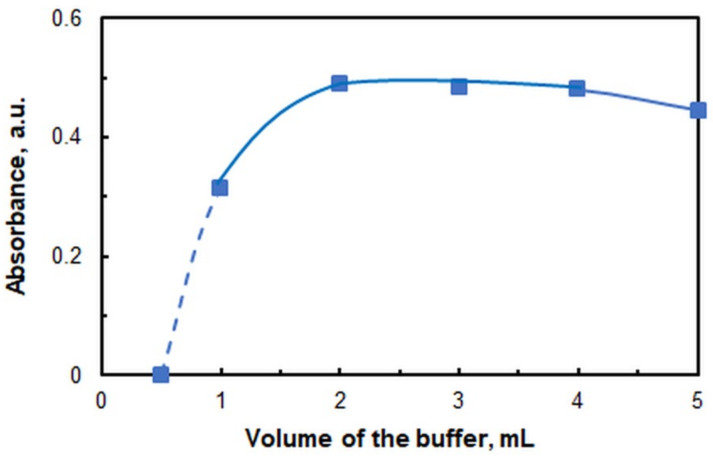
Effects of buffer volume. pH 5.5; *c*_V_ = 4.0 × 10^−6^ M; *c*_4NC_ = 7.5 × 10^−4^ M; *c*_CPC_ = 5 × 10^−4^ M; *w*_TX_ = 0.4%; *t*_inc_ = 70 min at 60 °C; λ = 670 nm.

The impact of the buffer volume is shown in Figure 3. The subsequent studies were conducted in the presence of 2.0 mL buffer. The employment of reduced buffer volumes is not recommended, as it is difficult to separate a surfactant-rich phase (SRP). In such cases, the application of centrifugal force is imperative.

**Figure 4:**
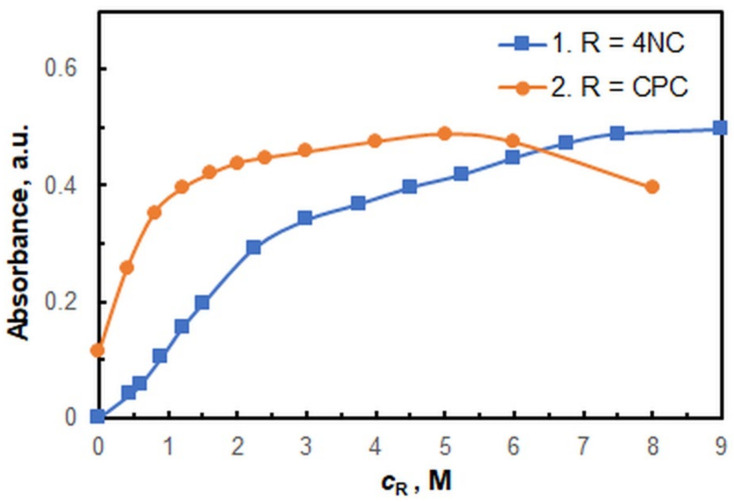
Effects of reagent concentrations. *c*_V_ = 4 × 10^−6^ M; *w*_TX_ = 0.4%; pH = 5.5; *t*_inc_ = 70 min at 60 °C; λ = 670 nm. *c*_CPC_ = 5 × 10^−4^ M (curve 1); *c*_4NC_ = 7.5 × 10^−4^ M (curve 2).

The effects of reagent concentrations are shown in Figure 4. The optimal 4NC concentration is 7.5 × 10^−4^ M (curve 1), and the optimal CPC concentration is 5.0 × 10^−4^ M (curve 2). The utilization of more concentrated 4NC solutions is not recommended, as this renders the procedure more costly and may result in the partial reduction of V^V^ to V^IV^ [44].

**Figure 5:**
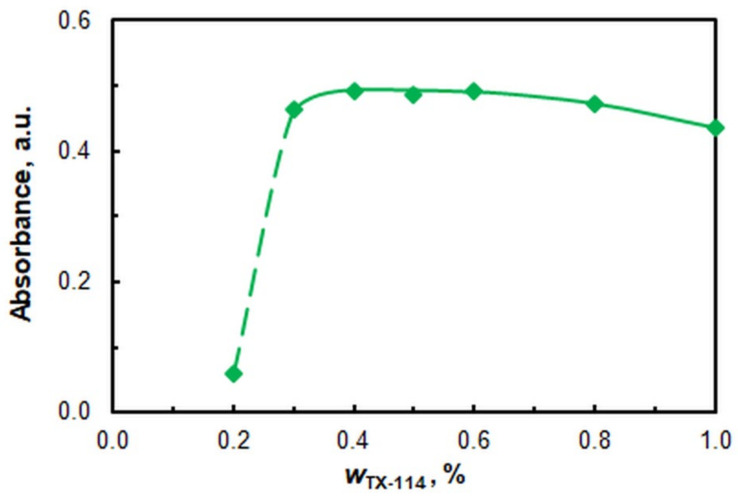
Effects of TX mass fraction. *c*_V_ = 4 × 10^−6^ M; *c*_4NC_ = 7.5 × 10^−4^ M; *c*_CPC_ = 5 × 10^−4^ M; pH = 5.5; *t*_inc_ = 70 min at 60 °C; λ = 670 nm.

The effects of the TX mass fraction (*w*_TX_) are displayed in Figure 5. Achieving a stable SRP necessitates a minimum *w*_TX_ of 0.3%. The subsequent studies were conducted at *w*_TX_ = 0.4%. This corresponds to a mass ratio of the two surfactants of approximately 1:22 (CPC:TX).

**Figure 6:**
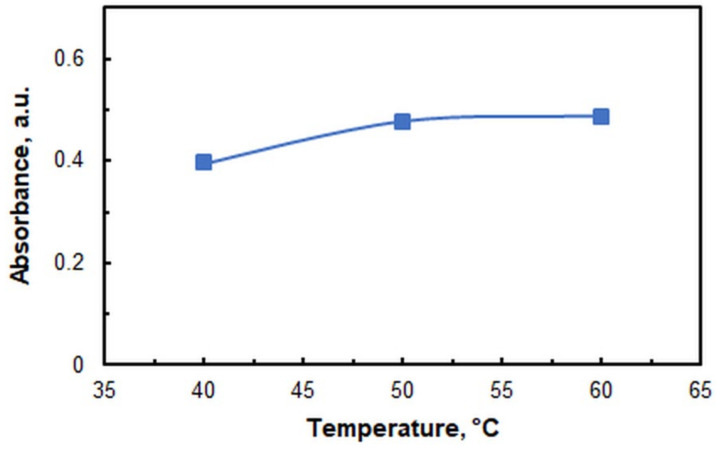
Effects of incubation temperature. *c*_V_ = 4 × 10^−6^ M; *c*_4NC_ = 7.5 × 10^−4^ M; *c*_CPC_ = 5 × 10^−4^ M; *w*_TX_ = 0.4%; pH = 5.5; *t*_inc_ = 70 min; λ = 670 nm.

**Figure 7:**
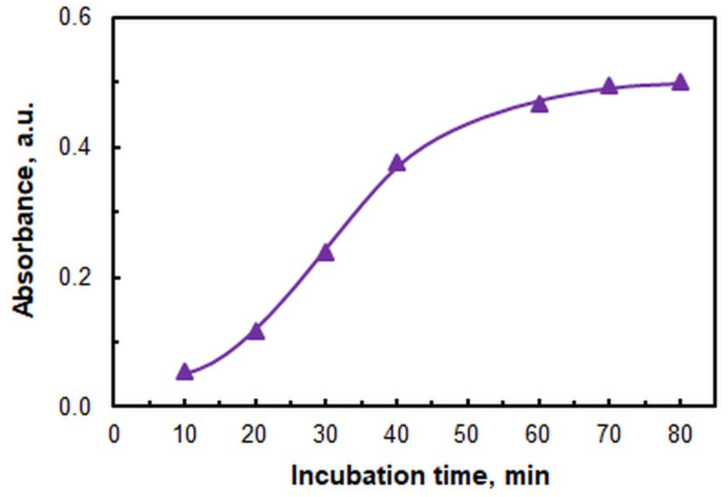
Effects of incubation time at 60 *°*C. *c*_V(V)_ = 4 × 10^−6^ M; *c*_4NC_ = 7.5 × 10^−4^ M; *c*_CPC_ = 5 × 10^−4^ M; *w*_TX_ = 0.4%; pH = 5.5; λ = 670 nm.

The influence of incubation temperature and incubation time (*t*_inc_) is illustrated in Figure 6 and Figure 7, respectively. The subsequent studies were conducted in a water bath set to 60 °C. The recommended incubation time, as deduced from Figure 7, is 70 min. The temporal measurement was initiated from the moment when the samples were submerged in the preheated (60 °C) bath.

The complete set of optimized parameters is enumerated in Table 1. The cooling time and SRP treatment are analogous to those described in our previous paper dealing with the Mo^VI^ complex [52].

### 2.2. Stoichiometry, Complex Formation Equation, and Extraction Constant

**Figure 8:**
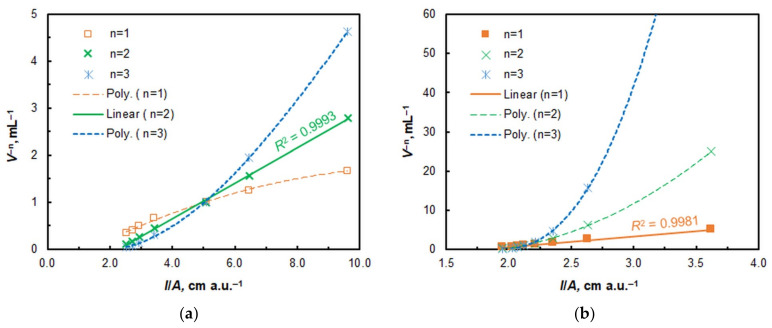
Determination of 4NC:V^V^ (**a**) and CPC:V^V^ (**b**) molar ratios by method of Asmus.

**Figure 9:**
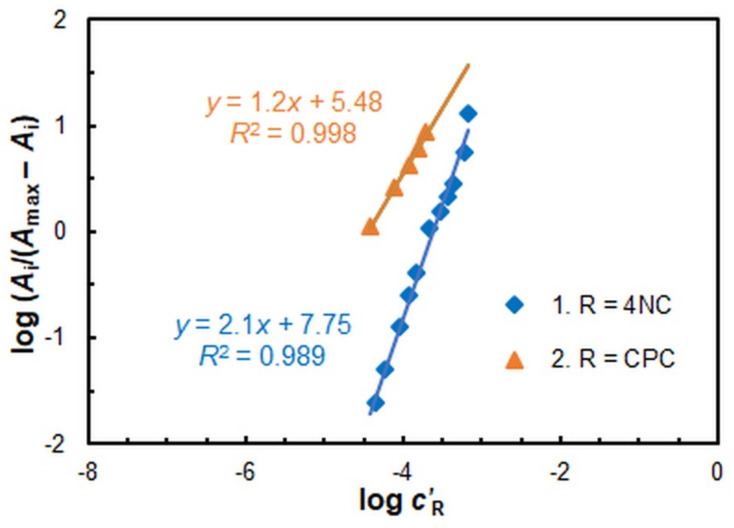
Determination of 4NC:V^V^ (straight-line 1) and CPC:V^V^ (straight-line 2) molar ratios by mobile equilibrium method.

The molar ratios between the components of the extracted complex were determined using the straight-line Asmus method [54] (Figure 8) and the mobile equilibrium method [55] (Figure 9). The findings from both methods (*n*_4NC_:*n*_V_ = 2:1 and *n*_CPC_:*n*_V_ = 1:1) suggest that the CL-CPE of the ternary complex can be represented by the following equation: 2H_2_L_(aq)_ + H_2_VO_4_^−^_(aq)_ + CP^+^_(aq)_ ≡ (CP^+^)[V^V^OL_2_]_(SRP)_ + 3H_2_O. This equation is consistent with the forms of the existence of V^V^ [2,56] and 4NC (H_2_L) [57,58,59] under the optimal conditions.

The conditional extraction constant (*K*_ex_) was calculated using the Holme–Langmyhr method [60] and the mobile equilibrium method [55]. The obtained logarithmic values demonstrate a high degree of agreement: Log*K*_ex_ = 4.67 ± 0.15 (± SD; *n* = 7, Holme–Langmyhr method) and Log*K*_ex_ = 4.57 ± 0.10 (± SD; *n* = 4, mobile equilibrium method).

### 2.3. Analytical Characteristics

A calibration graph was constructed under optimal conditions (see Table 1) using vanadium standards with concentrations ranging from 10 to 815 ng mL^−1^. The linear interval extends up to 305 ng mL^−1^ V^V^ (*n* = 9), and positive deviations from Beer’s law are observed at higher concentrations. The linear regression equation was found to be *A* = 2.40*γ* − 0.004, where *A* is the absorbance, and *γ* is the V^V^ concentration (µg mL^−1^). The standard deviations of the slope and intercept were 0.02 and 0.004, respectively. The apparent molar absorption coefficient, which corresponds to the slope, was determined to be 1.22 × 10^5^ M^−1^ cm^−1^. The Sandell sensitivity was 0.42 ng cm^−2^. The limits of detection and quantitation were 0.6 ng mL^−1^ and 2 ng mL^−1^, respectively. These values were calculated as three and ten times the standard deviation (SD) of the blank (4.8 × 10^−4^, *n* = 10) divided by the slope. A preconcentration factor of 16.4 was found by dividing the maximum sample volume (50 mL) by the volume of the diluted SRP (*V*_SPR_ = 3.05 mL). *V*_SPR_ was determined using the mass of the final solution (3.00 g) and its density (measured with a pycnometer).

The absorbance of the complex demonstrates high stability over time. A 20 h experiment was conducted to evaluate this stability. The results, expressed in terms of calibration curve parameters, are presented in Table 2. As can be seen, the changes are negligible during the first 2–3 h. It is only after 20 h that the slope of the calibration plot increases slightly, by approximately 6%. This increase can be attributed to the evaporation of the solvent or the reduction of V^V^ to V^IV^. It is noteworthy that throughout the experiment, the linearity of the calibration plot remained perfect, and the intercept was statistically indistinguishable from zero.

### 2.4. Effect of Diverse Ions

The effect that diverse ions have on the determination of V^V^ is demonstrated in Table 3. Substantial quantities of common ions, such as alkali and alkaline earth ions, Cl^−^, Br^−^, NO_3_^−^, and SO_4_^2−^, do not elicit interference. Many transition metal ions, including Cd^II^, Co^II^, Hg^II^, Mn^II^, Ni^II^, and Zn^II^, also do not interfere. However, a 250-fold excess of Pb^II^, a 100-fold excess of U^VI^, and a 5-fold excess of Cr^VI^ cause the sample to become turbid, thereby obstructing the analysis. Mo^VI^, W^VI^, and Cu^II^ form extractable complexes [52,61], but their absorption bands are situated at shorter wavelengths. A 20-fold excess of Mo^VI^ and 50-fold excesses of W^VI^ and Cu^II^ are considered tolerable. The most significant interferences are induced by Al^III^, Fe^III^, and Cr. The incorporation of NaF effectively mitigated the adverse effect caused by Al^III^. The same masking agent possesses the capacity to extinguish interference from minor excesses of Fe^III^. In such instances, the measurement of light absorption should be conducted at a longer wavelength (e.g., 720 nm). The elimination of Cr^III^ interference can be achieved by masking with sodium citrate and recording the absorbance at 700 or 720 nm. The measurement at these wavelengths results in sensitivity reductions of 5% and 11%, respectively.

### 2.5. Analytical Application

The novel method was tested in the analysis of mineral water samples, a spent Monsanto LP-110 catalyst, and a vanadium-containing dietary supplement (Chromium & Vanadium, Natural Factors^®^, Canada).

The mineral water analysis results are outlined in Table 4.

The analysis of the spent catalyst yielded a vanadium content of (2.72 ± 0.03)% and an RSD of 1.1% (*n* = 4). This content was corroborated by an alternative LLE method [62] (2.76 ± 0.07)%.

The dietary supplement was analyzed in the presence of sodium citrate, as it contains chromium. The analytical result obtained was 24.8 µg of vanadium per capsule, with an RSD of 1% (*n* = 4). This value was statistically indistinguishable from the manufacturer’s declared value of 25 µg.

### 2.6. Comparison with Other Methods and Practicality Assessment

Table 5 provides a comparative analysis of the present method’s key characteristics with those of other spectrophotometric extraction methods. This method is characterized by its simplicity, convenience, robustness, repeatability, selectivity, budget-effectiveness, and ecological friendliness. Unlike conventional CPE methods [19,24,31,43,63], a centrifuge is not necessary because of spontaneous phase separation. Adding a salting-out agent to increase extraction efficiency, as outlined in the method in [43], is also unnecessary. In contrast to the methods [24,33,39] that use a micro-syringe or spatula for the final phase isolation, the present method offers a simpler and faster approach: decantation. The reagents, 4NC and CPC, are inexpensive and widely available. No synthesis of reagents is required, as is the case with the methods in [24,35,64]. The calibration plot demonstrates excellent linearity, and the absorption remains stable over time. Furthermore, the blank’s absorbance is negligible and can be disregarded for most applications. This streamlines processing and boosts the method’s productivity.

**Table 5:** Comparison with other extractive spectrophotometric methods for vanadium determination.

Reagent(s)	Extraction Technique	Extractant	Linear Range, ng mL^−1^	*λ*_max_, nm	*ε*·10^−4^, L mol^−1^ cm^−1^	LOD, ng mL^−1^	Sample	Ref.
Br-PADAP	CPE	TX-114	7–300	603	NR	2.2	Tap water and river water	[31]
DCDHNAQ	SPE	Carbon-18	10–450	573	24.5	3.2	Water, alloy, soil, and urine	[64]
DPs + HAs	LLE	Chloroform	400–16,000	614–630	2.95–3.85	9	Soil, water, and food samples	[40]
EDTA + Safranin T	UA-CPE	TX-114	2–180 (V^V^) 1–40 (V^IV^)	530	NR	0.53 (V^V^) 0.26 (V^IV^)	Vegetal oils and vinegar	[63]
HTAR + H_2_O_2_	CPE	TX-100	Up to 510	582	16.6	0.8	Lake water and spent catalysts	[36]
HTAR + TTC	LLE	Chloroform	15–2000	549	5.2	4.6	Spent catalysts and pharmaceuticals	[62]
HTAR + NTC	LLE	Chloroform	23–1100	556	5.2	6.8	–	[62]
*N*-BPHA	LLE	Chloroform	0–1500	530	0.545	NR	Natural waters	[37]
NTA8HQ + BIABP + H_2_O_2_	CPE	TX-114	1–70 (V^V^) 10–100 (V^IV^)	634 625	160 (V^V^) 206 (V^IV^)	0.72 (V^V^) 1.78 (V^IV^)	Water, soil, rice, and vegetables	[35]
PAR	MCPE	TX-114	50–600	568	1.85	5.51	Tap water	[33]
PAR + BDTA	SPE	Clinoptilolite	10–3000	550	NR	0.07	Synthetic samples and alloys	[65]
PG + Safranin T	UA-CPE	TX114	2–500	533	NR	0.58	Beverages	[43]
TA + CTAB	DLLME-SFOD	1-Undecanol	6–1000	600	NR	1.8	Fruit juice samples	[39]
TAN + H_2_O_2_	CL-CPE	TX-100	Up to 760	607	8.84	1.4	Natural water, aluminum alloy, catalyst, and solution for infusion	[32]
TAR + H_2_O_2_	CL-CPE	TX-100	Up to 510	569	7.4	1.7	Dietary supplement and catalysts	[34]
4NC + MTT	LLE	Chloroform	120–1200	400	3.13	35	Steel and catalysts	[45]
4NC + CPC	MM-CL-CPE	TX114	2–305	670	12.2	0.6	Mineral water, dietary supplement, and spent catalyst	This work

Abbreviations: 4NC, 4-nitrocatechol; BDTA, benzyldimethyltetradecyleammonium chloride; BIABP, 2-[(benzoimidazolyl)azo]-4-benzyl phenol; Br-PADAP, 2-(5-bromo-2-pyridylazo)-5-diethylaminophenol; CL-CPE, centrifuge-less CPE; CPC, cetylpyridinium chloride; DCDHNAQ, 2,3-dichloro-6-(2,7-dihydroxy-1-naphthylazo)quinoxaline; DLLME-SFOD, dispersive liquid–liquid microextraction based on solidified floated organic drop; DPs, dithiolphenoles; HAs, hydrophobic amines; HTAR, 6-hexyl-4-(2-thiazolylazo)-resorcinol; LLE, liquid–liquid extraction; MCPE, micro-CPE; MM-CL-CPE, mixed micelle centrifuge-less CPE; MTT, 3-(4,5-dimethyl-2-thiazol)2,5- diphenyltetrazolium bromide; *N*-BPHA, *N*-benzoyl-*N*-phenylhydroxylamine; NR, not reported; NTA8HQ, 2-[2-(5-nitrothiazolyl)azo]-8-hydroxyquinoline; PAR, 4-(2-pyridylazo)resorcinol; PG, pyrogallol; SPE, solid-phase extraction; TAN, 1-(2-thiazolylazo)naphthol; TAR, 4-(2-thiazolylazo)resorcinol; TX-100, Triton X-100; TX-114, Triton X-114; UA-CPE, ultrasonic-assisted CPE.

A potential drawback of the current CL-CPE approach is the relatively long heating and cooling times. However, if necessary, the cooling time can be reduced by following the standard CPE procedures of centrifugation and ice bath application [19,24,31,43,63] without compromising sensitivity and selectivity. The heating process can likely be expedited through the use of a microwave oven, as evidenced in [66]. However, further research and optimization would be required to implement this approach. It is evident that a reduction in these times would result in other concomitant drawbacks, such as heightened labor requirements and the necessity for additional equipment.

**Figure 10:**
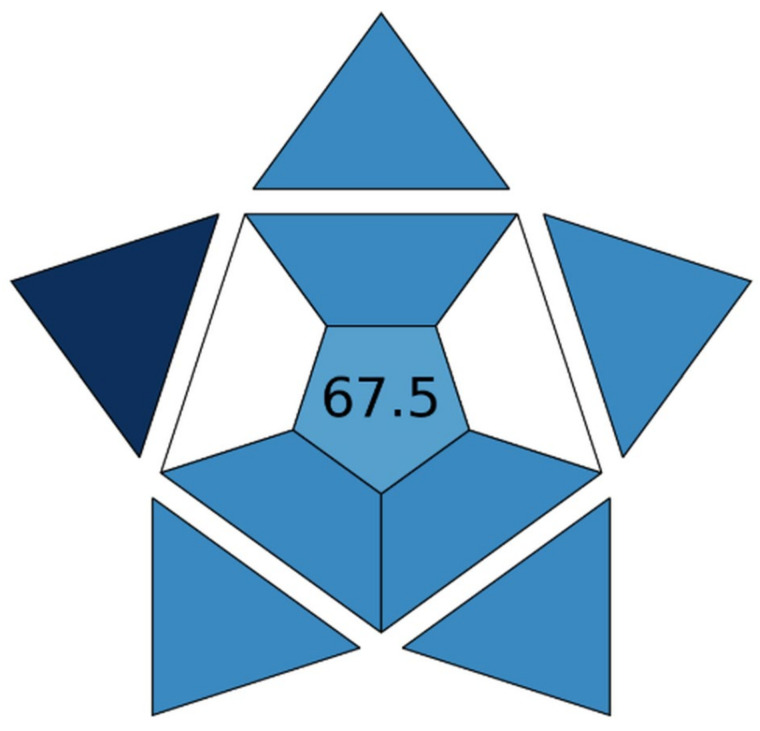
BAGI pictogram for proposed method.

Despite the aforementioned slow processes, this novel method can be considered “practical”. The validity of this conclusion is reinforced by the findings presented in Figure 10, which indicate a score of 67.5 on the blue applicability grade index (BAGI) [67].

## 3. Materials and Methods

### 3.1. Reagents and Chemicals

The chemicals were procured from Merck (Schnelldorf, Germany) and utilized without further purification as aqueous solutions. The V^V^ standard solution was prepared from ammonium metavanadate (puriss. p.a.) at a concentration of 2.0 × 10^−4^ M. Solutions of 4NC (≥96.0%) and CPC (cetylpyridinium chloride monohydrate, ≥96.0%) were prepared at concentrations of 1.875 × 10^−2^ M and 2.50 × 10^−2^ M, respectively. The mass fraction of the laboratory-grade TX solution was 10%. Buffer solutions with a pH range of 4.5–9.1 were prepared by blending 2.0 M solutions of ammonia and acetic acid. Water was purified by either deionization (18.2 MΩ cm) or distillation.

### 3.2. Instrumentation

The UV-Vis spectrophotometer used was the Ultrospec 3300 pro (Garforth, UK). The instrument was outfitted with 10 mm macro-cuvettes, with a capacity of 2.5 mL. The CPE experiment utilized an Ohaus Pioneer PA214C top-loading analytical balance (Parsippany, NJ, USA) and a GFL 1023 (Berlin, Germany) water bath. The pH measurements were conducted using a WTW InoLab 7110 pH meter (Weilheim, Germany).

### 3.3. Samples and Sample Preparation

Bottled mineral water (Gorna Banya^®^, Sofia, Bulgaria) was procured from a local supermarket. Another source of mineral water was extracted directly from the spring in the town of Bratsigovo, Bulgaria. The analysis was conducted on the following day. Aliquots of 30 mL were used for each of these analyses.

The spent catalyst (Monsanto LP-110), employed in the production of H_2_SO_4_, was obtained from Holding KCM 2000, Plovdiv, Bulgaria. The preparation for analysis was carried out in accordance with the established procedure [62]. Aliquots of 0.5 mL were utilized in the CPE step.

The dietary supplement, Chromium & Vanadium (Natural Factors^®^, Coquitlam, BC, Canada), was obtained from a local pharmacy. According to the product’s specifications, each capsule should contain 25 μg of vanadium (citrate) and 100 μg of chromium (GTF chelate). The formulation also contains microcrystalline cellulose, gelatin, and vegetable-grade magnesium stearate. The following procedure was implemented to dissolve the capsules. First, the gelatin shells were removed, and the contents were transferred to a beaker. Then, 15–20 mL of 0.1 M hydrochloric acid was added and gently heated to approximately 40 °C for 30 min. The mixture was then filtered through an FN 2 paper filter, and the filtrate was collected in a 25 mL volumetric flask. The oxidation of V^IV^ to V^V^ was achieved by the addition of several drops of 0.02 M KMnO_4_. The pink color should be visible for 1–2 min. Finally, 2 mL of ethanol was added to reduce any Cr^VI^, and the flask was filled to the mark with 0.1 M HCl.

### 3.4. Optimization Procedure

Solutions of TX, buffer, 4NC, V^V^, and CPC were transferred to pre-weighed, 50 mL conical-bottom centrifuge tubes. The mixtures were diluted with water to 50 mL and then incubated in a water bath at 40–60 °C for 10–80 min. The tubes were then cooled briefly under running water and stored in a freezer set to –20 °C for 40–60 min. This finalized the precipitation process and facilitated the removal of the upper layer through decantation. Subsequently, 0.5 mL of ethanol and a few drops of water were added to the viscous SRP, yielding a total mass of 3.00 g for each sample. Finally, the mixtures were homogenized and loaded into cuvettes to measure the corresponding absorbances.

### 3.5. Recommended Procedure for Determining Vanadium

Place an aliquot of the analyzed solution containing no more than 305 ng mL^−1^ V^V^ in a 50 mL conical-bottom tube. Then add 2.0 mL of 10% TX solution, 2.0 mL of pH 5.5 buffer solution, 2.0 mL of a 1.875 × 10^−2^ M 4NC solution, and 1.0 mL of a 2.5 × 10^−2^ M CPC solution. If necessary, add a masking agent solution (sodium citrate or sodium fluoride). Dilute the sample to 50 mL with water, then place it in a water bath at 60 °C. After 70 min of incubation, cool the sample under running water, then put it in a freezer set at −20 °C for 55 min. Decant the top layer after that. Add 0.5 mL of ethanol to the SRP, then add enough water to bring the total mass to 3.00 g. Homogenize the mixture, then load the cuvette. Measure the absorbance against water at a wavelength of 670 nm. When masking Cr^III^ with citrate, measurements at 700 or 720 nm are recommended. Finally, calculate the unknown V^V^ concentration from a calibration curve.

## 4. Conclusions

A novel spectrophotometric method using CPE was proposed in this study for determining vanadium. It can be classified as a mixed micelle-mediated, centrifuge-less method, for which the acronym MM-CL-CPE is appropriate. The method’s merits were demonstrated through real-sample analyses and the implementation of the BAGI metric tool. The method was proven to be simple, convenient, robust, repeatable, sensitive, selective, cost-effective, and eco-friendly—in short, “practical”. This novel method has the potential to compete effectively with other methods for the analysis of total vanadium. One of its primary benefits is the negligible blank absorption, which leads to significant analytical advantages.

## Figures and Tables

**Table 1:** Optimal operating conditions.

Parameter	Optimal Value
Wavelength, nm	670
pH	5.5
Volume of buffer, mL	2.0
Concentration of 4NC, M	7.5 × 10^−4^
Concentration of CPC, M	5.0 × 10^−4^
Mass fraction of TX, %	0.4
Incubation time, min	70
Incubation temperature, °C	60
Cooling time (at −20 °C), min	55
Test tube capacity, mL	50
Mass * of diluted SRP, g	3.00

* In the presence of water and 0.5 mL C_2_H_5_OH.

**Table 2:** Changes in calibration plot * parameters over time.

Time	Calibration Plot Parameters		
	Slope ± SD	Intercept ± SD	*R* ^2^
0 min = 30 min	2.402 ± 0.026	–0.0045 ± 0.0049	0.9998
60 min	2.401 ± 0.025	–0.0044 ± 0.0048	0.9998
120 min	2.400 ± 0.024	–0.0043 ± 0.0047	0.9998
200 min	2.417 ± 0.023	–0.0040 ± 0.0044	0.9998
20 h	2.549 ± 0.015	–0.0013 ± 0.0028	0.9999

* Calibration plots, including the zero-point, were constructed for V^V^ concentrations of 0.10, 0.20, and 0.30 μg mL^−1^ (two replicates).

**Table 3:** The effect of foreign ions on the determination of 10.2 μg V^V^.

Ion	Added Salt Formula	Ion–V(V) Mass Ratio	V(V) Found, μg	*R*, %
Al^III^	Al_2_(SO_4_)_3_·18H_2_O	5	9.3	91.6
		100 ^a^	10.0	98.4
Ba^II^	Ba(NO_3_)_2_	2000 ^b^	10.3	101
Br^−^	NaBr	2000 ^b^	9.9	97.6
Ca^II^	Ca(NO_3_)_2_	2000 ^b^	10.6	104
Cd^II^	Cd(NO_3_)_2_·4H_2_O	2000 ^b^	9.6	94.4
Citrate^3−^	Na_3_C_6_H_5_O_7_	500	10.0	98.3
		2000	9.4	92.2
Cl^−^	NaCl	2000 ^b^	10.4	102
Co^II^	CoSO_4_·7H_2_O	500	10.0	98.1
Cr^III^	Cr_2_(SO_4_)_3_	2	11.3	110
		5 ^c,d^	10.1	99.2
Cr^VI^	K_2_CrO_4_	1	10.7	105
		2	11.1	109
Cu^II^	Cu(SO_4_)_2_·5H_2_O	50	10.4	102
F^−^	NaF	500	9.8	96.2
Fe^III^	Fe_2_(SO_4_)_3_	1	12.3	121
		2 ^a,d^	10.2	100
Hg^II^	Hg(NO_3_)_2_	500	9.8	96.1
HPO_4_^2−^	Na_2_HPO_4_·12H_2_O	1000	10.0	98.5
I^−^	KI	100	10.4	102
Mg^II^	MgSO_4_·7H_2_O	2000 ^b^	9.7	95.6
Mn^II^	MnSO_4_·5H_2_O	1000	10.0	98.4
Mo^VI^	Na_2_MoO_4_	20	10.4	102
Ni^II^	NiSO_4_·7H_2_O	2000 ^b^	10.0	98.5
Pb^II^	Pb(NO_3_)_2_	100	10.2	100
Re^VII^	NH_4_ReO_4_	75	10.1	99.5
Sr^II^	Sr(NO_3_)_2_	2000 ^b^	10.1	99.4
Tartrate^2−^	KNaC_4_H_4_O_6_·4H_2_O	1000	10.1	99.0
U^VI^	UO_2_(CH_3_CO_2_)_2_·H_2_O	50	9.4	92.4
W^VI^	Na_2_WO_4_·2H_2_O	50	10.7	105
Zn^II^	ZnSO_4_·7H_2_O	750	10.0	98.3

^a^ In the presence of F^−^ (5.1 mg). ^b^ Higher mass ratios were not studied. ^c^ In the presence of citrate^3−^ (5.1 mg). ^d^ Measured at λ = 720 nm.

**Table 4:** Determination of V^V^ in unspiked and spiked mineral water samples.

Mineral Water	V^V^ Concentration, ng mL^−1^		RSD, %	*R*, %
	Added	Found ^c^		
Sample 1 ^a^	0	Not detected	–	–
	20.4	21.0 ± 0.3	1.4	103.3
	40.8	39.7 ± 0.8	2.0	97.4
	61.1	61.6 ± 0.3	0.5	100.8
Sample 2 ^b^	0	2.1 ± 0.2	8.0	–
	20.4	20.9 ± 0.6	1.9	92.4
	40.8	43.7 ± 1.5	1.9	102.0
	61.1	63.2 ± 3.3	6.5	100.0

^a^ Commercial bottled mineral water. ^b^ Mineral water extracted directly from spring. ^c^ ±SD, *n* = 3.

## Data Availability

Data are contained within the article.
